# Machine Learning-Predicted Progression to Permanent Atrial Fibrillation After Catheter Ablation

**DOI:** 10.3389/fcvm.2022.813914

**Published:** 2022-02-16

**Authors:** Je-Wook Park, Oh-Seok Kwon, Jaemin Shim, Inseok Hwang, Yun Gi Kim, Hee Tae Yu, Tae-Hoon Kim, Jae-Sun Uhm, Jong-Youn Kim, Jong Il Choi, Boyoung Joung, Moon-Hyoung Lee, Young-Hoon Kim, Hui-Nam Pak

**Affiliations:** ^1^Division of Cardiology, Yonsei University Health System, Seoul, South Korea; ^2^Department of Internal Medicine, Korea University Cardiovascular Center, Seoul, South Korea

**Keywords:** atrial fibrillation, catheter ablation, progression, machine learning, risk score

## Abstract

**Introduction:**

We developed a prediction model for atrial fibrillation (AF) progression and tested whether machine learning (ML) could reproduce the prediction power in an independent cohort using pre-procedural non-invasive variables alone.

**Methods:**

Cohort 1 included 1,214 patients and cohort 2, 658, and all underwent AF catheter ablation (AFCA). AF progression to permanent AF was defined as sustained AF despite repeat AFCA or cardioversion under antiarrhythmic drugs. We developed a risk stratification model for AF progression (STAAR score) and stratified cohort 1 into three groups. We also developed an ML-prediction model to classify three STAAR risk groups without invasive parameters and validated the risk score in cohort 2.

**Results:**

The STAAR score consisted of a stroke (2 points, *p* = 0.003), persistent AF (1 point, *p* = 0.049), left atrial (LA) dimension ≥43 mm (1 point, *p* = 0.010), LA voltage <1.109 mV (2 points, *p* = 0.004), and PR interval ≥196 ms (1 point, *p* = 0.001), based on multivariate Cox analyses, and it had a good discriminative power for progression to permanent AF [area under curve (AUC) 0.796, 95% confidence interval (CI): 0.753–0.838]. The ML prediction model calculated the risk for AF progression without invasive variables and achieved excellent risk stratification: AUC 0.935 for low-risk groups (score = 0), AUC 0.855 for intermediate-risk groups (score 1–3), and AUC 0.965 for high-risk groups (score ≥ 4) in cohort 1. The ML model successfully predicted the high-risk group for AF progression in cohort 2 (log-rank *p* < 0.001).

**Conclusions:**

The ML-prediction model successfully classified the high-risk patients who will progress to permanent AF after AFCA without invasive variables but has a limited discrimination power for the intermediate-risk group.

## Introduction

Atrial fibrillation catheter ablation (AFCA) is known to be effective for rhythm control management, which can improve symptoms and quality of life in atrial fibrillation (AF) patients (1). Recent study and guideline reported a beneficial effect of AFCA on mortality and heart failure hospitalization in AF patients with left ventricular dysfunction (1, 2). Although there is a substantial recurrence rate after AFCA, a positive clinical impact can be expected from a reduction in AF burden itself unless sustained AF continues after a procedure (3, 4). Recurrence after AFCA is defined as AF or atrial tachycardia (AT) of 30 s or more regardless of symptoms (1). At this point, although it is important to reduce a recurrence rate of atrial arrhythmias lasting 30 s, it might also be very important to identify progression to permanent AF in which it is difficult to control sustained AF even after repeated procedures or with use of antiarrhythmic drugs (AADs). By predicting progression to permanent AF before a *de novo* AFCA procedure, we can select patients who are not likely to progress to permanent AF, thereby reducing any unnecessary risk and cost and potentially improving a success rate of a procedure. Therefore, pre-discovery of patients who are likely to progress to permanent AF using pre-procedural parameters may contribute to improvements in AFCA rhythm and clinical outcomes. However, many peri-procedural predictors, including both non-invasive and invasive parameters, contribute in a complex manner to AFCA rhythm outcomes (5). So variable studies reported or validated the risk model for AF recurrence in the patients who underwent repeat ablations (5). Those studies showed the range of ACU from 0.487 to 0.833. However, only a study reported risk model derived from over 1,000 patient population (6), but the follow-up duration was relatively short of investigating the progression to permanent AF (mean 2.5 years). Based on a variety of sources of complex patient information, including electronic medical records (EMRs) and imaging data, artificial intelligence (AI) has been used to detect AF and to predict ablation outcomes (7–12). Furthermore, AI has been suggested for predicting invasive parameters or invasive cardiovascular outcomes (13).

In this retrospective analysis of prospective AFCA cohort data, we aimed to develop a machine learning (ML)-prediction model to identify patients with progression to permanent AF using the pre-procedural non-invasive factors. As an intermediate step for developing the ML prediction model, we used a risk score derived from this study population and identified the high-risk group for progression to permanent AF. Therefore, we developed and used an ML prediction model to classify the patients into risk score-based groups associated with progression to permanent AF.

## Methods

### Study Population

**Figure 1:**
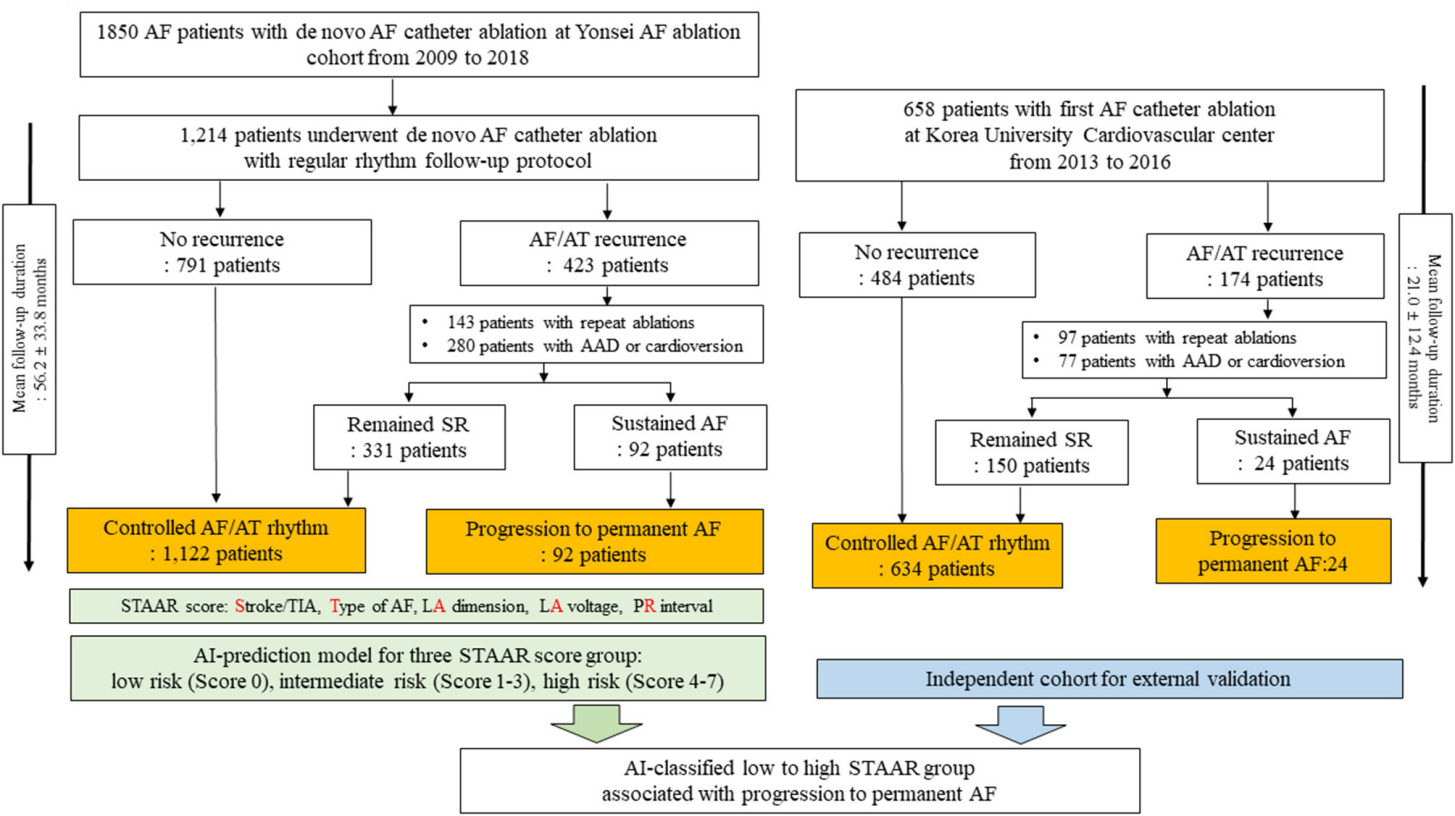
Study flow chart. AF, atrial fibrillation; AT, atrial tachyarrhythmia; SR, sinus rhythm; LA, left atrium; AAD, antiarrhythmic drugs.

This study protocol adhered to the principles of the Declaration of Helsinki and was approved by the Institutional Review Board of the Yonsei University Health System. All patients provided written informed consent for inclusion in the Yonsei AF Ablation Cohort Database (registered at ClinicalTrials.gov, Identifier: NCT02138695). Figure 1 shows the enrollment into this study. A total of 1,850 patients who underwent AFCA from 2009 to 2018 were registered in the Yonsei AF ablation cohort. The cohort was adjusted based on the following exclusion criteria: (1) permanent AF refractory to electrical cardioversion, (2) AF with rheumatic valvular disease, or any mechanical or bioprosthetic heart valve, (3) prior cardiac surgery with concomitant AF surgery or AF catheter ablation, and (4) the absence of a mean left atrial (LA) voltage that could be obtained during the *de novo* ablation procedure. Finally, 1,214 patients {mean age: 58.7 ± 10.9 years, 26.5% female, 31.4% persistent AF, median follow-up duration [interquartile range (IQR)]: 51.4 months (26.9, 83.7)} were enrolled and used to develop risk score and ML prediction models in this study.

Among the 1,214 patients in the development data, 791 remained in sinus rhythm after the first AFCA during the follow-up period [median 38.5 months (23.9, 70.7)]. Among the 423 patients who experienced AF/AT recurrences after the first AFCA, 143 underwent repeat ablation sessions and 280 underwent electrical cardioversion under AADs to restore sinus rhythm. We considered the progression to permanent AF, which is the endpoint of this study, as sustaining AF detected on an electrocardiogram (ECG) or Holter/event-monitor after last ablation procedures with AADs or electrical cardioversion. For example, the patient was considered as progression to permanent AF group when the rhythm status was AF in an ECG or Holter-monitoring at last visit of outpatient clinics on AADs for recurred AF after the last ablation procedures. We defined the controlled arrhythmia group as consisting of those patients who experienced no AF/AT recurrences or intermittent AF/AT after the repeat ablation sessions, AADs, or electrical cardioversion during the follow-up period. Finally, we obtained the following groups from the development data: 1,122 patients with controlled arrhythmias and 92 with progression to permanent AF.

### Electrocardiographic, Echocardiographic, and Cardiac Computed-Tomography Evaluations

In order to develop a risk model, we used 12-lead ECG, in which the sinus rhythm and PR interval can be identified, that was be obtained most recently before AFCA, as previously described (14). In brief, the 12-lead ECG of all patients (GE Healthcare, Marquette, MAC5500, Waukesha, WI) was obtained in this study. The paper speed was 25 mm/s and calibration 10 mm/mV. All patients underwent transthoracic echocardiography before the *de novo* procedure. We obtained the echocardiographic parameters according to the American Society of Echocardiography guidelines. The patients underwent three-dimensional (3D) spiral CT scans (64 Channel, LightSpeed Volume CT, Philips, Brilliance 63, Amsterdam, Netherlands) to define the pulmonary vein (PV) anatomy. The 3D spiral CT images of the LA were analyzed on an imaging processing workstation (Aquarius, TeraRecon Inc., Foster City, California, United States).

### Electrophysiological Mapping and Radiofrequency Catheter Ablation

AFCA was performed by using a 3D electroanatomical mapping system (NavX, St. Jude Medical, Inc., Minnetonka, Minnesota, United States; CARTO, Biosense-Webser, Inc., Diamond Bar, California, United States). We conducted multi-view pulmonary venograms after the transseptal puncture. The 3D electroanatomical geometry of both the LA and PVs was constructed and merged with the 3D spiral CT images. An LA voltage map was obtained during sinus rhythm using bipolar electrograms from 350 to 500 points on the LA endocardium during atrial pacing at 500 ms, and the mean LA voltage was calculated according to previous reports (15, 16). The LA voltage data was obtained in all patients of the dependent cohort. If the initial rhythm was AF before the ablation procedure, we restored sinus rhythm using internal electrical cardioversion. If the AF was frequently reinitiated after recurrent electrical cardioversion before the ablation procedure, we obtained LA voltage map after the ablation procedure.

An open-irrigated tip catheter (Celsius, NaviStar ThermoCool, ThermoCool SF, and ThermoCool SmartTouch, Johnson & Johnson Inc., Diamond Bar, California, United States; CoolFlex, FlexAbility, and TactiCath, St. Jude Medical Inc., Minnetonka, Minnesota, United States) was used for the AFCA. All patients underwent a *de novo* procedure with a circumferential pulmonary vein isolation (CPVI). The majority of the patients (91.8%) underwent a cavotricuspid isthmus (CTI) block procedure during the *de novo* procedure unless there was atrioventricular conduction disease. We added additional linear ablation such as a roof line, posterior inferior line (posterior box lesion), or anterior line, especially in patients with persistent AF. A left lateral isthmus ablation, right atrial ablation, and complex fractionated electrogram ablation were performed in a minority of the patients at the operator's discretion. We ended the *de novo* procedure when there was no immediate recurrence of AF within 10 min after the cardioversion with an isoproterenol infusion (5–10 μg/min depending on ß-blocker use and target sinus heart rate of 120 bpm). In the case of mappable AF triggers or premature atrial beats, the extra-PV foci were carefully mapped and ablated as much as possible. Systemic anticoagulation was achieved using intravenous heparin to maintain an activated clotting time of 350–400 s during the procedure.

### Post-ablation Management and Follow-Up

Patients visited an outpatient clinic at 1, 3, 6, and 12 months and every 6 months thereafter or whenever symptoms developed after AFCA. Patients underwent an ECG at every visit. Twenty-four-hour Holter monitoring was performed at 3, 6, and 12 months and then every 6 months after the AFCA according to the 2012 Heart Rhythm Society/European Heart Rhythm Association/European Cardiac Arrhythmia Society expert consensus statement guidelines. Whenever the patients suffered from symptoms of palpitations, Holter/event-monitor examinations were performed to investigate the possibility of an arrhythmia recurrence. We defined an AF/AT recurrence as any episode of AT or AF lasting for 30 s or more. Any electrocardiography documentation of an AF recurrence after a 3-month blanking period was classified as a clinical recurrence.

### Management of Recurrence and Progression to Permanent AF

Patients who experienced AF/AT recurrences after the first AFCA were prescribed AADs or electrical cardioversion based on the rhythm condition. If a recurrent atrial arrhythmia could not be controlled by medications, we recommended a repeat ablation to the patients and their family members. We previously reported the detailed technique and strategy for repeat ablation procedures (17). In brief, if there were reconnections of the PV potentials, a CTI or additional linear lines that were created in the index procedure, we first completed them as much as possible. Then, we provoked extra-PV foci with an isoproterenol infusion (5–10 μg /min) and carefully mapped and ablated any mappable AF triggers or frequent atrial premature beats.

After the repeat ablation procedures, we performed a serial rhythm follow-up, adhering to the above-mentioned protocol. We defined patients with progression to permanent AF as those patients who remained in a sustained AF/AT rhythm at the final follow-up date despite a repeat ablation, AADs, or electrical cardioversion.

### Risk Score for Progression to Permanent AF: STAAR Score

We performed Cox regression analysis using clinical variables to develop the risk score for progression to permanent AF. First, we decided on the optimal cut-off values for the LA dimension (43 mm), PR interval (196 ms), and mean LA voltage (<1.109 mV) by using the Youden index. The predictors with *p*-values < 0.05 in the univariate Cox regression model and age were included in the multivariate Cox regression model without any stepwise methods. Finally, five predictors including persistent AF at baseline, a history of a stroke or TIA, an enlarged LA (≥43 mm), a prolonged PR interval (≥196 ms), and a low mean LA voltage (<1.109 mV) were associated with progression to permanent AF (Supplementary Table 1). Then, the STAAR score was developed using the following five predictors: a previous history of a stroke or transient ischemic attack (TIA, S), type of AF (T), LA dimension (A), LA voltage (A), and PR interval (R). The STAAR score was calculated based on the beta coefficients, which were divided by the smallest absolute value of the regression coefficient and rounded to the nearest integer. And each patient was calculated by summing the points assigned to each predictor (Supplementary Table 2): a previous history of a stroke or TIA (2 points), the AF type (1 point for persistent AF at the time of the diagnosis), LA size (1 point for an enlarged LA ≥ 43 mm), mean LV voltage (2 points for a low mean LA voltage <1.109 mV), and PR interval (1 point for a prolonged PR interval ≥ 196 ms).

### Random Forest for Developing the ML-Prediction Model to Classify Three STAAR Risk Groups

Using the median and interquartile value of STAAR score in the overall population, the patients with individual STAAR scores were divided into three STAAR risk groups which had each risk level for progression to permanent AF; low risk (score 0) for progression to permanent AF, intermediate-risk (score 1–3) for progression to permanent AF, and high risk (score 4–7) for progression to permanent AF. Because the STAAR score needed the invasive parameter such as LA voltage, we developed an ML-prediction model for predicting three STAAR risk groups for progression to permanent AF using non-invasive parameters. For a risk stratification model of AF progression to permanent AF (STAAR score), we developed a model based on the Random Forest (RF) (18) and implemented the software with a Scikit-learn library (version 1.0.1). The input parameters (15 × 1) used non-invasive clinical features [age, female sex, AF type, body mass index (BMI), congestive heart failure (CHF), hypertension, diabetes mellitus, stroke or TIA, vascular disease, LA dimension, left ventricular ejection fraction (LVEF), Eem [peak transmitral flow velocity (E) divided by tissue Doppler echocardiography of the peak septal mitral annular velocity (Em)], creatinine, hemoglobin, and pre-ablation PR interval]. Before hyperparameter selection by cross-validation, the training and test sets were randomly divided at an 8:2 ratio. Then, an optimal model was induced with 20-fold cross-validation from the training set (0.8) and the predictive performance of the RF classifier was evaluated with the test set (0.2). However, it may still induce overlooked biases as the data sets provided for cross-validation may not be representative of patient or population diversity (19). Therefore, we performed the nested cross-validation with five “outer” test sets consisting of 5 iterations. Finally, we identify the best RF classifier with this nested cross-validation procedure. The output *Y*_*class*_ consisted of three classes and each class was divided into the following criteria:


$$\begin{array}{l} Y_{class}(z)=\{\begin{array}{cc} Low-risk\;group, & z<1\\ Intermediate-risk\;group, & 1\leq z\leq 3\\ High-risk\;group, & z>3 \end{array}, \end{array}$$

where z is the STAAR score, and *Y*_*class*_ has a higher risk when it increases from 0 to 2. Hyperparameters for optimal performance of the RF model were carefully selected by grid search algorithm (20) using the GridSearchCV function of the Scikit-learn library. The selected hyperparameters are bootstrap: true, the maximum depth of the tree (max_depth: 8), the number of features to consider when looking for the best split (max_feature: 5), the minimum number of samples required to be at a leaf node (min_samples_leaf: 4), the minimum number of samples required to split an internal node (min_samples_split: 10), and the number of trees in the forest (n_estimators: 100). The selected hyperparameters and search ranges are shown in Supplementary Table 3. Supplementary Figure 1 shows an example of a trained first decision tree. To check for overfitting (using the Scikit-learn library version 1.0.1), the results using a method of High Energy Physics showed that the effect of the class imbalance is not significant with the distance of the points (test set) to the bars (training set) (Supplementary Figure 2). For the reproducibility, all model training was performed for five iterations with a random sample.

### Independent AFCA Cohort to Predict the Ablation Outcome

For external validation of the ML-prediction model to classify three STAAR risk groups, we used an independent AF ablation cohort that included 805 patients who underwent their first AFCA at Korea University Cardiovascular Center between 2013 and 2016. From this independent data, we enrolled and analyzed 658 patients who were classified into three risk groups for progression to permanent AF based on the ML-prediction model that classified three STAAR risk groups. Among the 658 patients in the independent cohort, 484 remained in sinus rhythm after the first AFCA during the follow-up period. Of the 174 patients who experienced an AF/AT recurrences after the first AFCA, 97 underwent repeat ablation sessions and 77 underwent AADs or electrical cardioversion during the follow-up period. Finally, we identified 634 patients as the controlled arrhythmia group and 24 as the progression to permanent AF group for the external validation. There were significant size and follow-up duration discrepancies between training and external validation cohorts, but the ablation procedure data of two cohorts were similar to each other.

### Statistical Analysis

Continuous variables are reported as means ± standard deviations (SD) and were analyzed using an independent *t*-tests. Categorical variables are reported as numbers (percentages) and were analyzed using a Chi-square or Fisher's exact test. We defined a cut-off value of the LA dimension, PR interval, and LA voltage using the Youden index. The Youden index decides a cut-off point at the specific value where the amount of sensitivity plus specificity was maximal value (21, 22). As a result, the Youden index yielded the highest value of the following equation at the specific point: sensitivity + specificity-1. To develop STAAR risk model, we included the predictors with *p*-values < 0.05 in the univariate Cox regression analysis and age in the multivariate Cox regression analysis without any stepwise methods. CHA2DS2VASc score was excluded in multivariate Cox regression analysis because the score already included age, previous history of congestive heart failure, and stroke/TIA (Supplementary Table 1). We confirmed the proportional hazard assumption of the STAAR risk model using log-minus-log Kaplan-Meier plot. Linear regression analysis was performed to investigate the relationship between pre-procedural factors and the STAAR score (Supplementary Table 4). The ANOVA test for continuous variables or a Chi-square or Fisher's exact test for categorical variables were performed to investigate characteristics among three STAAR risk groups before developing the ML-risk model to classify three STAAR risk groups (Supplementary Table 5). An independent *t*-test for continuous variables or a Chi-square or Fisher's exact test for categorical variables were used to investigate the difference in baseline characteristics between the controlled AF/AT group and progression to permanent AF group in the independent cohort (Supplementary Table 6). The STAAR score performance was assessed with a C-statistic representing the area under the curve (AUC) of the receiver operating characteristic (ROC) curve. Because the STAAR score included invasive parameters, the ML prediction model was developed based on the RF algorithm using non-invasive parameters to classify the patients into three risk groups for progression to permanent AF. A Kaplan-Meier analysis was used to investigate the freedom from progression to permanent AF in the three STAAR groups of the development cohort and the three ML-predicted risk groups in the independent cohort. A *p*-value < 0.05 was considered statistically significant. All statistical analyses were performed using SPSS (Statistical Package for Social Sciences, Chicago, Illinois, United States) software for Windows (version 23.0) and the R package (3.1.0, R Foundation for Statistical Computing, Boston, Massachusetts, United States).

## Results

### Baseline Characteristics

The comparison of the baseline characteristics between the controlled AF/AT group (*n* = 1,122; 92.4%) and progression to permanent AF group (*n* = 92, 7.6%) is presented in Table 1. Patients with progression to permanent AF had a higher proportion of baseline persistent AF (*p* < 0.001), higher BMI (*p* = 0.039), higher number of previous stroke or TIA events, and longer PR interval than the controlled AF/AT group. In terms of the echocardiographic parameters, the LA dimension (*p* < 0.001) was larger in the progression group than in the rhythm-controlled group. The invasive parameters acquired during *de novo* ablation procedures showed that the LA voltage was lower (*p* < 0.001), ablation time was longer (*p* = 0.036), and extra-PV LA ablation more commonly performed (*p* = 0.001) in the progression group than its counterpart (Table 2). In the independent cohort with median 18.9 (IQR: 11.4, 30.3) months of follow-up, mean age was 56.8 ± 10.7 years, the proportion of female was 20.8%, and the proportion of persistent AF was 40.3%.

**Table 1:** Baseline characteristics during the *de novo* ablation procedure.

	**Overall patients (*n* = 1,214)**	**Controlled AF/AT (*n* = 1,122)**	**Progression to permanent AF (*n* = 92)**	***p*-value**
Age, years	58.7 ± 10.9	58.6 ± 11.0	60.1 ± 10.0	0.193
Female	322 (26.5%)	298 (26.6%)	24 (26.1%)	0.921
Persistent AF at diagnosis	381 (31.4%)	325 (29.0%)	56 (60.9%)	**<0.001**
Body mass index, kg/m^2^	25.0 ± 3.2	25.0 ± 3.2	25.7 ± 3.1	**0.039**
CHA_2_DS_2_VASc score	1.7 ± 1.6	1.7 ± 1.5	2.0 ± 1.7	0.053
CHF	126 (10.4%)	114 (10.2%)	12 (13.0%)	0.383
Hypertension	575 (47.4%)	527 (47.0%)	48 (52.2%)	0.336
Diabetes mellitus	176 (14.5%)	161 (14.3%)	15 (16.3%)	0.609
Stroke/TIA	143 (11.8%)	123 (11.0%)	20 (21.7%)	**0.002**
Vascular disease	150 (12.4%)	142 (12.7%)	8 (8.7%)	0.267
LA dimension, mm	41.3 ± 6.1	41.0 ± 6.0	45.2 ± 5.9	**<0.001**
LVEF, %	63.3 ± 8.4	63.4 ± 8.3	62.2 ± 9.2	0.208
EEm (*n* = 1,159)	10.2 ± 4.2	10.1 ± 4.1	10.9 ± 5.2	0.111
Creatinine, mg/dL	0.9 ± 0.3	0.9 ± 0.3	0.9 ± 0.3	0.737
Hemoglobin, g/dL	14.4 ± 1.5	14.4 ± 1.5	14.5 ± 1.3	0.355
Pre-ECG PR interval, ms	184.0 ± 31.8	182.6 ± 29.7	201.4 ± 40.6	**<0.001**

*AF, atrial fibrillation; AT, atrial tachyarrhythmia; CHF, congestive heart failure; TIA, transient ischemic attack; LA, left atrium; LVEF, left ventricular ejection fraction; Eem, peak transmitral flow velocity (E), and tissue Doppler echocardiography of the peak septal mitral annular velocity (Em); ECG, electrocardiography. The bold values represents p-value < 0.05*.

**Table 2:** Ablation characteristics and outcomes during the *de novo* ablation procedure.

	**Overall patients (*n* = 1,214)**	**Controlled AF/AT (*n* = 1,122)**	**Progression to permanent AF (*n* = 92)**	***p*-value**
Mean LA voltage, mV	1.3 ± 0.6	1.3 ±0.6	0.9 ± 0.5	**<0.001**
Ablation time, min	81.4 ± 27.5	81.0 ± 27.3	87.3 ± 29.3	**0.036**
Procedure time, min	181.2 ± 53.3	180.2 ± 52.7	193.2 ± 58.7	**0.025**
Contact force sensing catheter	95 (7.8%)	89 (7.9%)	6 (6.5%)	0.628
**Extra-PV LA ablation**	442 (36.4%)	393 (35.1%)	49 (53.3%)	**0.001**
Roof line	436 (36%)	387 (34.6%)	49 (53.3%)	**<0.001**
Postero-inferior line	373 (30.8%)	334 (29.8%)	39 (42.4%)	**0.012**
Left latera isthmus	48 (4.0%)	42 (3.8%)	6 (6.5%)	0.171
Anterior line	325 (26.8%)	283 (25.2%)	42 (45.7%)	**<0.001**
**Bidirectional block of linear ablation**				
Roof line	378/436 (86.7%)	334/387 (86.3%)	44/49 (86.3%)	0.498
Postero-inferior line	218/373 (58.4%)	202/334 (60.5%)	16/39 (41%)	**0.020**
Left lateral isthmus line	10/48 (20.8%)	10/42 (23.8%)	0 (0%)	0.320
Anterior line	231/325 (65.5%)	187/283 (66.1%)	26/42 (61.9%)	0.595
CFAE ablation	63 (5.2%)	54 (4.8%)	9 (9.8%)	**0.049**
Post-ablation AAD use	194 (16%)	161 (14.4%)	33 (35.9%)	**<0.001**
**Type of AAD after recurrence (*****n*** **=** **249)**				
Class Ic drug	90 (36.1%)	73 (38.2%)	17 (29.3%)	0.216
Class III drug	159 (63.9%)	118 (61.8%)	41 (70.7%)	
Number of repeat ablations (in 143 patients)	2.0 ± 0.3 (*n* = 143)	2.1 ± 0.3 (*n* = 118)	2.0 ± 0.4 (*n* = 25)	0.203
Duration between 1st and 2nd AFCA, months (*n* = 143)	35.7 ± 26.6	36.4 ± 26.8	31.8 ± 25.5	0.443
AF/AT recurrence after repeat ablations	55/143 (38.5%)	30/118 (25.4%)	25 (100%)	**<0.001**
Progression to permanent AF after repeat ablations	25/143 (17.5%)	0 (0%)	25 (100%)	**<0.001**

*AF, atrial fibrillation; AT, atrial tachyarrhythmia; PV, pulmonary vein; LA, left atrium; CFAE, complex fractionated atrial electrogram; AAD, antiarrhythmic drugs; AFCA, AF catheter ablate. The bold values represents p-value < 0.05*.

### Association Between the STAAR Score and Progression to Permanent AF

**Figure 2:**
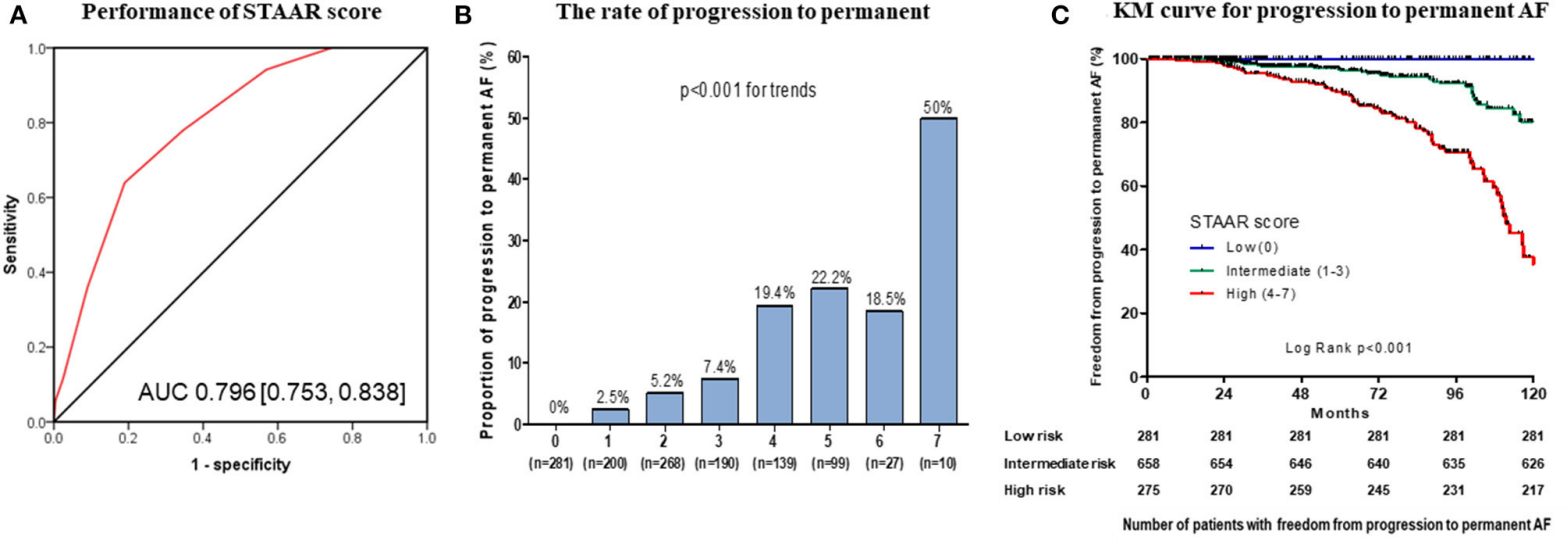
Performance and Kaplan-Meier (KM) analysis of the STAAR score. Performance of the STAAR score for the discrimination of the progression to permanent AF **(A)**. The rate of progression to permanent AF according to the STAAR score **(B)**. KM analysis with censor marker (black vertical line) of the progression to permanent AF according to the STAAR group **(C)**. AF, atrial fibrillation; AUC, area under the curve.

In the development data, an AUC of ROC curve of the STAAR score, which showed the discriminative power for progression to permanent AF, was 0.796 (95% CI: 0.753–0.838, Figure 2A). The STAAR score ranged from 0 to 7 points. There was a significantly increased proportion of patients who progressed to permanent AF in those with a higher risk score (0% at a risk score of 0 to 50% at a risk score of 7, *p*-value for the trend <0.001, Figure 2B). The patients were classified into three risk groups which had each risk level for progression to permanent AF: low (0 points), intermediate (1–3 points), and high (>3 points). The population and baseline characteristics in the three risk groups are presented in Supplementary Table 5. The high-risk group for progression to permanent AF was older (*p* < 0.001), had more proportion of comorbidities, more enlarged LA (*p* < 0.001), and more prolonged PR interval on ECG before ablation (*p* < 0.001) as compared to low and intermediate-risk group. The high-risk group for progression to permanent AF had a significantly greater progression to permanent AF than the low-risk and intermediate-risk groups for progression to permanent AF in the Kaplan-Meier analysis (log-rank *p* < 0.001, Figure 2C).

### ML-Prediction Model Predicting the Progression to Permanent AF

**Figure 3:**
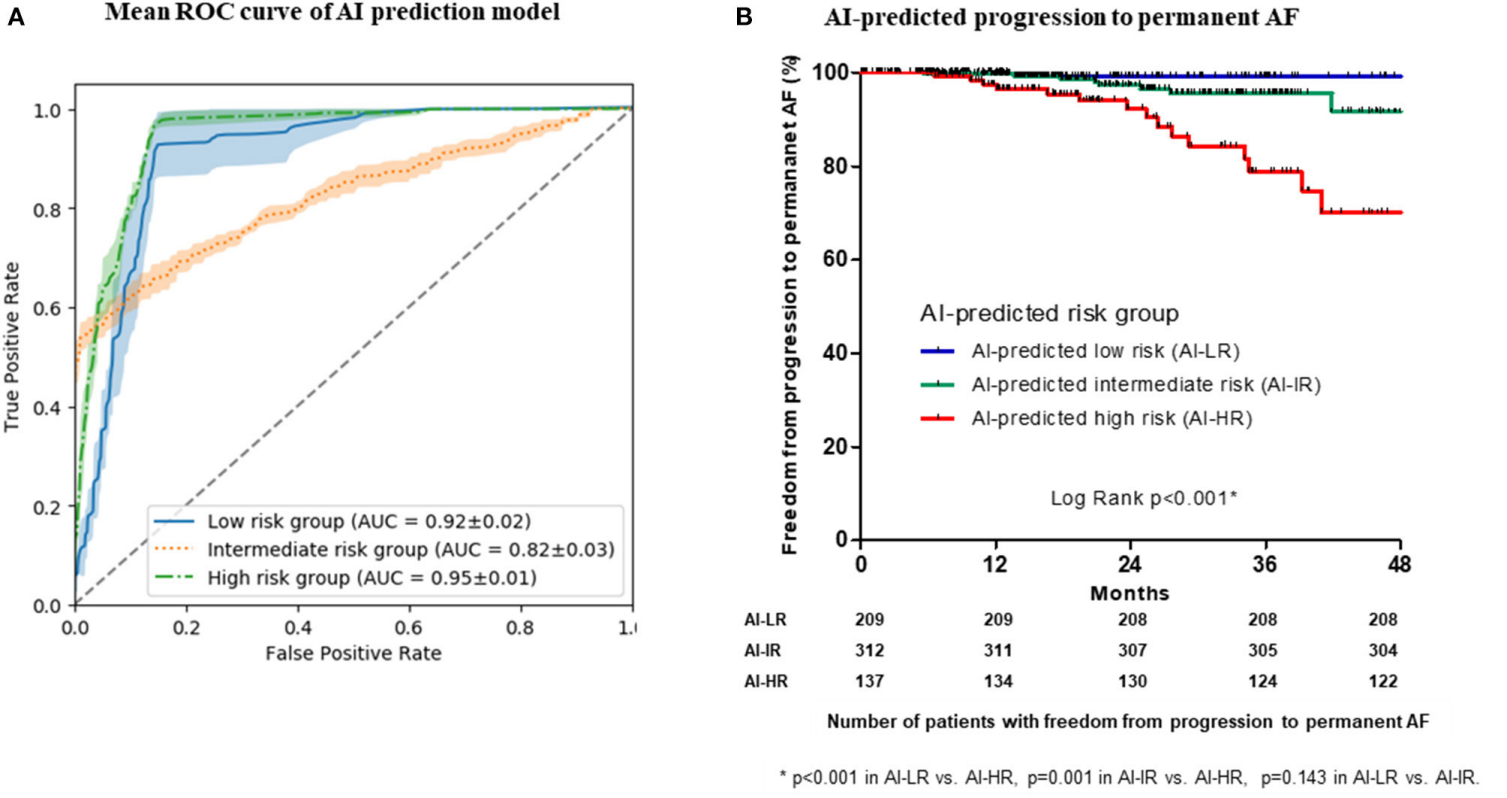
The mean area under the curve (AUC) of the receiver operating characteristic (ROC) curves of the machine learning (ML) prediction model to classify three STAAR risk groups **(A)**. Kaplan-Meier analysis with censor marker (black vertical line) of the progression to permanent AF according to the ML-predicted risk groups in the independent cohort **(B)**. AF, atrial fibrillation.

Using pre-procedural non-invasive variables (age; female sex; AF type; BMI; comorbidities including CHF, hypertension, diabetes mellitus, stroke or TIA, vascular disease; LA dimension; LVEF; EEm creatinine; hemoglobin; and pre-ablation PR interval), we developed an ML prediction model to stratify the risk for the progression to permanent AF in cohort 1. We chose those 15 variables based on a linear regression analysis without multicollinearity among the variables (Supplementary Table 4). The training and test set were derived from randomly selected samples in each test. All tests were performed five times. The mean ROC curves of the five tests, which had a discriminative power for the three risk groups for progression to permanent AF, are presented in Figure 3A. The performance of the five ML prediction models to classify three STAAR risk groups is presented in Table 3. Among the five randomly tested models, we chose the best prediction model, Test 4 in Supplementary Table 7 (performance predicting low risk: AUC 0.935, sensitivity 0.945, specificity 0.847; performance predicting intermediate risk: AUC 0.855, sensitivity 0.717, specificity 0.848; and performance predicting high risk: AUC 0.965, sensitivity 0.960, specificity 0.890) for the verification in the independent cohort 2. In the independent cohort 2, the ML prediction model using pre-procedural non-invasive variables successfully classified the three risk groups for progression to permanent AF, and the patients in the ML-predicted high-risk group had a significantly higher rate of progression to permanent AF than those in the low-risk (log-rank *p* < 0.001, Figure 3B) and the intermediate-risk (log-rank *p* = 0.001, Figure 3B) groups.

**Table 3:** Summary of prediction performance of the five machine learning-prediction model for the three STAAR groups in the development cohort^*^.

	**AUC**	**Sens**	**Spec**	**PPV**	**NPV**	**Gini**	**Loss**	**MSE**	**ACC**
LRG	0.923 (0.896, 0.935)	0.927 (0.927, 0.945)	0.842 (0.836, 0.847)	0.646 (0.638, 0.658)	0.974 (0.974, 0.980)	0.846 (0.791, 0.870)	0.862 (0.858, 0.871)	0.923 (0.896, 0.935)	0.927 (0.927, 0.945)
IRG	0.828 (0.786, 0.852)	0.717 (0.661, 0.724)	0.800 (0.705, 0.848)	0.814 (0.752, 0.850)	0.697 (0.661, 0.706)	0.656 (0.572, 0.704)	0.759 (0.724, 0.772)	0.828 (0.786, 0.852)	0.717 (0.661, 0.724)
HRG	0.946 (0.943, 0.950)	0.940 (0.900, 0.960)	0.857 (0.846, 0.874)	0.649 (0.616, 0.671)	0.981 (0.969, 0.987)	0.892 (0.885, 0.901)	0.879 (0.858, 0.888)	0.946 (0.943, 0.950)	0.940 (0.900, 0.960)

* *Each value of each risk group is presented as median (interquartile range) among five prediction model*.

*AI, articial intelligence; LRG, low risk group; IRG, intermediate risk group; HRG, high risk group; AUC, area under the curve; Sens, sensitivity; Spec, specificity; PPV, positive predictive value; NPV, negative predictive value; Gini, gini coefficient; Loss, logit loss; MSE, mean squared error; ACC, accuracy*.

## Discussion

### Main Findings

In this study, we investigated that the high STAAR score group (4–7) had a higher rate of progression to permanent AF after AFCA. However, the STAAR score was derived from an invasive parameter that could be obtained during the procedure. Using STAAR score as a labeling criterion, an ML prediction model based on a RF algorithm was developed to classify patients into risk groups for progression to permanent AF using non-invasive pre-AFCA variables. ML-predicted high-risk patients had a higher rate of progression to permanent AF than non-high-risk patients in an independent cohort.

### Mechanisms of AF Progression After AFCA

As a mechanism of recurrent AF after AFCA, technical factors such as an incomplete or inefficient ablation and disease factors such as substrate remodeling can be considered. Among the technical factors, there are operator factors and limitations of the current ablation technology. The operator-dependent variability of ablation success has been gradually decreasing due to development of contact force catheter technology and software such as an ablation index, lesion index, and one-shot technologies including cryoballoon ablation (23, 24). However, reconnections of PV potentials are still commonly observed in patients with recurrent AF, and we need to recognize limitations of the current ablation technology in generating a permanent conduction block during AFCA (17, 25, 26).

Nevertheless, AF is a progressive disease, and substrate remodeling is an important mechanism of AF progression and a long-term AF recurrence. Sustained AF aggravates atrial structural and electrical remodeling itself, which leads to a persistent AF burden (27). Pre-existing atrial remodeling, including an atrial low voltage substrate (28), magnetic resonance imaging (MRI)-detected atrial fibrosis (29), and elevated atrial pressure (30), have been proven to have negative effects on the rhythm outcome after AFCA. The PR interval reflects atrial remodeling with electrophysiological heterogeneity and a conduction delay throughout the atrium, which results from atrial fibrosis and inflammation (31) The fact that patients without PV reconnections in the redo mapping more commonly have extra-PV triggers and a higher chance of recurrence after the repeat ablation suggests the important role of the atrial substrate as a mechanism of a long-term recurrence (17, 32).

### STAAR Score and ML Risk Model to Classify Three STAAR Risk Groups Compared to Other Risk Models

Several risk models have been developed for AF recurrence after AFCA (5). Among them, there are two types of models: non-invasive and invasive risk models. Non-invasive risk models, which consist of non-invasive predictors, are ALARMEc, APPLE, ATLAS, CAAP-AF, and HATCH. These non-invasive risk models have discriminative power of AUC from 0.44 to 0.75. Invasive risk models, which consist of invasive predictors, are BASE-AF2 and MB-LATER. The AUC of these invasive risk models are between 0.57 and 0.94. However, a primary endpoint was progression to permanent AF in the current STAAR score and ML model in contrast with AF recurrence in other previous risk models. Because of this difference in an ablation outcome, we could not directly compare the performance for predicting ablation outcomes in the current model and other risk models. Although the highest AUC of invasive risk model is 0.94, early recurrence is an essential predictor for both BASE-AF2 and MB-LATER. Early recurrence can be obtained after AF ablation and may be underestimated in the patients with asymptomatic AF recurrence.

As compared to the published risk model and studies including repeat ablations (5), the current risk model was developed in the largest AF population who had the longest follow-up duration (Supplementary Table 8). We developed the current risk model using an ML algorithm to predict the high-risk patients progress to permanent AF despite AFCA using non-invasive non-imaging parameters. Therefore, the current ML-risk model can quickly discriminate the high-risk patient who might progress to permanent AF before the invasive ablations or expensive cardiac imaging at the outpatient clinics.

### Prognostic Value of AI in Cardiovascular Disease

Few AI models have been reported in other studies to predict the rhythm outcome of AFCA. Shade et al. (10) reported an AI model combined with late gadolinium enhancement (LGE)-MRI images to assess a probability of AF recurrence during a median 1-year follow-up period in patients with paroxysmal AF. Firouznia et al. (33) and Liu et al. (34) suggested an AI model using CT images of the LA or PVs to predict AF recurrence after AF ablation (33) or non-PV triggers in paroxysmal AF patients without AF recurrence during a 1-year follow-up period (34). Budzianowski et al. (11) reported an AI model that investigated predictors of early AF recurrence after cryoballoon ablation in numerous EMR data. Image-based AI models for AF recurrence or AF triggers need complex preprocessing procedures because MRI and CT images should be adjusted for the AI algorithms (10, 33, 34). As shown in this study, the ML prediction model may identify patients who are poor responders or super-responders to the specific treatment modalities over the long-term.

In the present study, although the STAAR score could well predict patients with progression to permanent AF over the long-term follow-up period, the STAAR score included the invasive parameters that could be obtained during an ablation procedure. An ML prediction model to classify three STAAR risk groups, which used non-invasive variables that were obtained in EMR, could replace the STAAR score. Furthermore, the ML prediction model could classify the patients into three risk groups with long-term progression to permanent AF in the development cohort. In an independent cohort, it could identify patients at a high risk for progression to permanent AF.

### Limitations

This study was an observational cohort study from two centers that included patients who were referred for AF ablation. There might be a selection bias. Due to the long duration of enrollment and follow-up, catheter technology and ablation strategies including additional linear ablation and extra-pulmonary foci ablation have been changed. It should be considered that those changes might affect the ablation results. Due to the different time of enrollment and follow-up duration between development and independent cohorts, it should be considered for the differences in type of catheter, ablation lesion set, and AF recurrence between the two cohorts. However, we found a time discrepancy of enrollment in training cohort and validation cohort in the previous AI-related studies (33, 35). Although PR interval can be influenced by medication, such as β-blockers or calcium channel blockers, and their dose, specific medication information at the time of sinus rhythm ECG was not available in this cohort study. Because the cut-off values of this study were derived from the patients in this single-cohort database, the values could not be fully applied to a wider population of AF. The number of the patients with progression to permanent AF was small in the replication cohort compared with an overall large population. Because the current ML model was validated in the independent cohort with a small number of events, this model may not be applied to the generalized AF population. Although it is common to set the label with the ground truth in the development of ML model, we used a STAAR score which is a processed risk score model. Therefore, a prospective randomized clinical trial is going on to prove the robustness of our ML model (ClinicalTrials.gov, NCT04997824). Because of using a one-vs.-all strategy for three groups of STAAR scores (low, intermediate, and high), the intermediate-risk group showed a relatively low AUC and the limited classification power from the low and high groups (Supplementary Figure 3). Although the current ML prediction model to classify three STAAR risk groups has been faithfully verified with 20-fold cross-validation and an external test set, actual predictive performance may differ in actual clinical application and needs future prospective study. Since we could not obtain the exact duration of maintaining sinus rhythm, there might be some patients who experienced relatively less AF recurrence, even among patients with progression to permanent AF. Holter or ECG monitoring might be insufficient to detect the subclinical AF recurrence and the rate of progression to permanent AF could be underestimated. The assessment of the last rhythm status in this study was variable because of different tools and intermittent time intervals for checking rhythm status. So, the progression to permanent AF, the endpoint in this study, should not be the same meaning to continuous AF all day. Operator or technical factors that could occur during AFCA were excluded from the risk score developed in this study. Because the definition of permanent AF might vary depending on the medical environment or physicians, the number of patients with progression to permanent AF was small even in the high-risk group, and also it needed long-term follow-up periods to identify the high-risk patients for progression to permanent AF. Due to the strict criteria of insurance coverage for AFCA in Korea, physicians generally start AADs first, and then recommend a repeat ablation when a recurrent atrial arrhythmia cannot be controlled by medication. Moreover, ML outcome is dependent on the data quality and the amount of training set. Therefore, the outcome of this study cannot be generalized to all patients with AF.

## Conclusions

An ML-prediction model successfully classified three risky groups that showed different risk levels of progression to permanent AF for long-term periods before an ablation procedure. Using pre-procedural and non-invasive variables, the ML prediction model can identify patients at high risk for progression to permanent AF before an ablation procedure but has a limited discrimination power for the patients with intermediate-risk.

## Data Availability Statement

The raw data supporting the conclusions of this article will be made available by the authors, without undue reservation.

## Ethics Statement

The studies involving human participants were reviewed and approved by the Institutional Review Board of the Yonsei University Health System. The patients/participants provided their written informed consent to participate in this study.

## Author Contributions

J-WP, O-SK, H-NP, and JS designed the current study, performed data analysis, and wrote the manuscript. IH performed the data analysis. YK, HY, T-HK, J-SU, J-YK, JC, BJ, M-HL, and Y-HK contributed to organize the database. H-NP and JS interpreted and discussed the results. All authors contributed to manuscript revision and approved the final version of the manuscript.

## Funding

This work was supported by Grant (HI21C0011 to H-NP) from the Korea Health 21 R&D Project, Ministry of Health and Welfare and a Grant (NRF-2020R1A2B01001695) from the Basic Science Research Program run by the National Research Foundation of Korea (NRF).

## Conflict of Interest

The authors declare that the research was conducted in the absence of any commercial or financial relationships that could be construed as a potential conflict of interest.

## Publisher's Note

All claims expressed in this article are solely those of the authors and do not necessarily represent those of their affiliated organizations, or those of the publisher, the editors and the reviewers. Any product that may be evaluated in this article, or claim that may be made by its manufacturer, is not guaranteed or endorsed by the publisher.

## Supplementary Material

Supplementary Figure 1 Example of a trained first decision tree.

Supplementary Figure 2 Results of overfitting checks for three STAAR groups. **(A–C)** Each group was divided by a one-vs.-all strategy and identified by the frequency of predicted value in the training and test sets.

Supplementary Figure 3 Confusion matrix result of artificial intelligence model (Test 4) for three STAAR group.

Supplementary Table 1 Cox regression analysis of the pre-procedural variables for the progression to permanent AF.

Supplementary Table 2 Prediction model and risk scores for the progression to permanent AF (STAAR score).

Supplementary Table 3 Optimal hyperparameters selected by grid search.

Supplementary Table 4 Linear regression analysis of the pre-procedural variables associated with the STAAR score.

Supplementary Table 5 Baseline characteristics during the *de novo* ablation according STAAR risk groups.

Supplementary Table 6 Baseline characteristics during the *de novo* ablation procedure in the independent cohort.

Supplementary Table 7 Prediction performance of the ML-prediction model for the three STAAR groups in the development cohort.

Supplementary Table 8 The previous studies and risk model predicting AF recurrence in the patients with repeat ablations for AF (1–9).

## Abbreviations

AFCA: atrial fibrillation catheter ablation
AAD: antiarrhythmic drugs
RF: random forest
STAAR: risk model consisting of previous history of a stroke or transient ischemic attack, type of AF, left atrial dimension, left atrial voltage, PR interval
Eem: peak transmitral flow velocity (E), and tissue Doppler echocardiography of the peak septal mitral annular velocity (em)
ALARMEc: risk model consisting of type of AF, metabolic syndrome, eGFR, and normalized left atrial area
APPLE: risk model consisting of age, type of AF, eGFR, left atrial diameter, and left ventricular ejection farction
ATLAS: risk model consisting of age, sex, type of AF, current smoking, and indexed left atrial volume
CAAP-AF: risk model consisting of age, sex, type of AF, LAD, coronary artery disease, and number of antiarrhythmic drugs failed
HATCH: risk model consisting of age, heart failure, hypertension, chronic obstructive pulmonary disease, and stroke/transient ischemic attack
BASE-AF2: risk model consisting of type of AF, LAD, body mass index, current smoking, AF history, and early recurrence
MB-LATER: risk model consisting of sex, type of AF, LAD, early recurrence, and bundle branch block.

## References

[1] Hindricks G Potpara T Dagres N Arbelo E Bax JJ Blomström-Lundqvist C. 2020 ESC Guidelines for the diagnosis and management of atrial fibrillation developed in collaboration with the European Association for Cardio-Thoracic Surgery (EACTS). Eur Heart J. (2021) 42:373–498. 10.1093/eurheartj/ehaa945 32860505

[2] Marrouche NF Brachmann J Andresen D Siebels J Boersma L Jordaens L. Catheter ablation for atrial fibrillation with heart failure. N Engl J Med. (2018) 378:417–27. 10.1056/NEJMoa1707855 29385358

[3] Jin MN Kim TH Kang KW Yu HT Uhm JS Joung B. Atrial fibrillation catheter ablation improves 1-year follow-up cognitive function, especially in patients with impaired cognitive function. Circ Arrhythm Electrophysiol. (2019) 12:e007197. 10.1161/CIRCEP.119.007197 31442075

[4] Park JW Yang PS Bae HJ Yang SY Yu HT Kim TH. Five-year change in the renal function after catheter ablation of atrial fibrillation. J Am Heart Assoc. (2019) 8:e013204. 10.1161/JAHA.119.013204 31474174 PMC6755838

[5] Dretzke J Chuchu N Agarwal R Herd C Chua W Fabritz L. Predicting recurrent atrial fibrillation after catheter ablation: a systematic review of prognostic models. Europace. (2020) 22:748–60. 10.1093/europace/euaa041 32227238 PMC7203634

[6] Winkle RA Jarman JW Mead RH Engel G Kong MH Fleming W. Predicting atrial fibrillation ablation outcome: the CAAP-AF score. Heart Rhythm. (2016) 13:2119–25. 10.1016/j.hrthm.2016.07.018 27435586

[7] Attia ZI Noseworthy PA Lopez-Jimenez F Asirvatham SJ Deshmukh AJ Gersh BJ. An artificial intelligence-enabled ECG algorithm for the identification of patients with atrial fibrillation during sinus rhythm: a retrospective analysis of outcome prediction. Lancet. (2019) 394:861–7. 10.1016/S0140-6736(19)31721-0 31378392

[8] Bumgarner JM Lambert CT Hussein AA Cantillon DJ Baranowski B Wolski K. Smartwatch algorithm for automated detection of atrial fibrillation. J Am Coll Cardiol. (2018) 71:2381–8. 10.1016/j.jacc.2018.03.003 29535065

[9] Tison GH Sanchez JM Ballinger B Singh A Olgin JE Pletcher MJ. Passive detection of atrial fibrillation using a commercially available smartwatch. JAMA Cardiol. (2018) 3:409–16. 10.1001/jamacardio.2018.0136 29562087 PMC5875390

[10] Shade JK Ali RL Basile D Popescu D Akhtar T Marine JE. Preprocedure application of machine learning and mechanistic simulations predicts likelihood of paroxysmal atrial fibrillation recurrence following pulmonary vein isolation. Circ Arrhythm Electrophysiol. (2020) 13:e008213. 10.1161/CIRCEP.119.008213 32536204 PMC7375930

[11] Budzianowski J Hiczkiewicz J Burchardt P Pieszko K Rzezniczak J Budzianowski P. Predictors of atrial fibrillation early recurrence following cryoballoon ablation of pulmonary veins using statistical assessment and machine learning algorithms. Heart Vessels. (2019) 34:352–9. 10.1007/s00380-018-1244-z 30140958 PMC6510876

[12] Hung M Lauren E Hon E Xu J Ruiz-Negrón B Rosales M. Using machine learning to predict 30-day hospital readmissions in patients with atrial fibrillation undergoing catheter ablation. J Pers Med. (2020) 10:82. 10.3390/jpm10030082 32784873 PMC7564438

[13] Al'Aref SJ Anchouche K Singh G Slomka PJ Kolli KK Kumar A. Clinical applications of machine learning in cardiovascular disease and its relevance to cardiac imaging. Eur Heart J. (2019) 40:1975–86. 10.1093/eurheartj/ehy404 30060039

[14] Park J Kim TH Lee JS Park JK Uhm JS Joung B. Prolonged PR interval predicts clinical recurrence of atrial fibrillation after catheter ablation. J Am Heart Assoc. (2014) 3:e001277. 10.1161/JAHA.114.001277 25292186 PMC4323778

[15] Lin YJ Tai CT Kao T Chang SL Wongcharoen W Lo LW. Consistency of complex fractionated atrial electrograms during atrial fibrillation. Heart Rhythm. (2008) 5:406–12. 10.1016/j.hrthm.2007.12.009 18313599

[16] Park JH Pak HN Kim SK Jang JK Choi JI Lim HE. Electrophysiologic characteristics of complex fractionated atrial electrograms in patients with atrial fibrillation. J Cardiovasc Electrophysiol. (2009) 20:266–72. 10.1111/j.1540-8167.2008.01321.x 19175848

[17] Kim TH Park J Uhm JS Joung B Lee MH Pak HN. Pulmonary vein reconnection predicts good clinical outcome after second catheter ablation for atrial fibrillation. Europace. (2017) 19:961–7. 10.1093/europace/euw128 27256420

[18] Breiman L. Random forests. Mach Learn. (2001) 45:5–32. 10.1023/A:1010933404324

[19] Jamet B Morvan L Nanni C Michaud AV Bailly C Chauvie S. Random survival forest to predict transplant-eligible newly diagnosed multiple myeloma outcome including FDG-PET radiomics: a combined analysis of two independent prospective European trials. Eur J Nucl Med Mol Imaging. (2021) 48:1005–15. 10.1007/s00259-020-05049-6 33006656

[20] LaValle SM Branicky MS Lindemann SR. On the relationship between classical grid search and probabilistic roadmaps. Int J Rob Res. (2004) 23:673–92. 10.1177/0278364904045481

[21] Youden WJ. Index for rating diagnostic tests. Cancer. (1950) 3:32. 10.1002/1097-0142(1950)3:1<32::AID-CNCR2820030106>3.0.CO;2-3 15405679

[22] Schisterman EF Perkins NJ Liu A Bondell H. Optimal cut-point and its corresponding Youden Index to discriminate individuals using pooled blood samples. Epidemiology. (2005) 16:73–81. 10.1097/01.ede.0000147512.81966.ba 15613948

[23] Reddy VY Dukkipati SR Neuzil P Natale A Albenque JP Kautzner J. Randomized, controlled trial of the safety and effectiveness of a contact force-sensing irrigated catheter for ablation of paroxysmal atrial fibrillation: results of the tacticath contact force ablation catheter study for atrial fibrillation (TOCCASTAR) study. Circulation. (2015) 132:907–15. 10.1161/CIRCULATIONAHA.114.014092 26260733

[24] Chen S Schmidt B Bordignon S Perrotta L Bologna F Chun KRJ. Impact of cryoballoon freeze duration on long-term durability of pulmonary vein isolation: ICE Re-map study. JACC Clin Electrophysiol. (2019) 5:551–9. 10.1016/j.jacep.2019.03.012 31122376

[25] Kim TH Park J Uhm JS Kim JY Joung B Lee MH. Challenging achievement of bidirectional block after linear ablation affects the rhythm outcome in patients with persistent atrial fibrillation. J Am Heart Assoc. (2016) 5:3894. 10.1161/JAHA.116.003894 27792644 PMC5121491

[26] Nilsson B Chen X Pehrson S Kober L Hilden J Svendsen JH. Recurrence of pulmonary vein conduction and atrial fibrillation after pulmonary vein isolation for atrial fibrillation: a randomized trial of the ostial versus the extraostial ablation strategy. Am Heart J. (2006) 152:537.e1–8. 10.1016/j.ahj.2006.05.029 16923426

[27] Walters TE Nisbet A Morris GM Tan G Mearns M Teo E. Progression of atrial remodeling in patients with high-burden atrial fibrillation: implications for early ablative intervention. Heart Rhythm. (2016) 13:331–9. 10.1016/j.hrthm.2015.10.028 26484789

[28] Vlachos K Efremidis M Letsas KP Bazoukis G Martin R Kalafateli M. Low-voltage areas detected by high-density electroanatomical mapping predict recurrence after ablation for paroxysmal atrial fibrillation. J Cardiovasc Electrophysiol. (2017) 28:1393–402. 10.1111/jce.13321 28884923

[29] Marrouche NF Wilber D Hindricks G Jais P Akoum N Marchlinski F. Association of atrial tissue fibrosis identified by delayed enhancement MRI and atrial fibrillation catheter ablation: the DECAAF study. JAMA. (2014) 311:498–506. 10.1001/jama.2014.3 24496537

[30] Park J Joung B Uhm JS Young Shim C Hwang C Hyoung Lee M. High left atrial pressures are associated with advanced electroanatomical remodeling of left atrium and independent predictors for clinical recurrence of atrial fibrillation after catheter ablation. Heart Rhythm. (2014) 11:953–60. 10.1016/j.hrthm.2014.03.009 24607916

[31] Hocini M Sanders P Deisenhofer I Jaïs P Hsu LF Scavée C. Reverse remodeling of sinus node function after catheter ablation of atrial fibrillation in patients with prolonged sinus pauses. Circulation. (2003) 108:1172–5. 10.1161/01.CIR.0000090685.13169.07 12952840

[32] Lin WS Tai CT Hsieh MH Tsai CF Lin YK Tsao HM. Catheter ablation of paroxysmal atrial fibrillation initiated by non-pulmonary vein ectopy. Circulation. (2003) 107:3176–83. 10.1161/01.CIR.0000074206.52056.2D 12821558

[33] Firouznia M Feeny AK LaBarbera MA McHale M Cantlay C Kalfas N. Machine learning-derived fractal features of shape and texture of the left atrium and pulmonary veins from cardiac CT scans are associated with risk of recurrence of atrial fibrillation post-ablation. Circ Arrhythm Electrophysiol. (2021) 14:e009265. 10.1161/CIRCEP.120.009265 33576688 PMC8015207

[34] Liu CM Chang SL Chen HH Chen WS Lin YJ Lo LW. The clinical application of the deep learning technique for predicting trigger origins in patients with paroxysmal atrial fibrillation with catheter ablation. Circ Arrhythm Electrophysiol. (2020) 13:e008518. 10.1161/CIRCEP.120.008518 33021404

[35] Feeny AK Rickard J Trulock KM Patel D Toro S Moennich LA. Machine learning of 12-lead QRS waveforms to identify cardiac resynchronization therapy patients with differential outcomes. Circ Arrhythm Electrophysiol. (2020) 13:e008210. 10.1161/CIRCEP.119.008210 32538136 PMC7901121

