# Supplementary figures and images for: Machine Learning-Predicted Progression to Permanent Atrial Fibrillation After Catheter Ablation
Source: Front Cardiovasc Med. 2022 Feb 16;9:813914. doi: 10.3389/fcvm.2022.813914 (PMC8890475; doi:10.3389/fcvm.2022.813914)

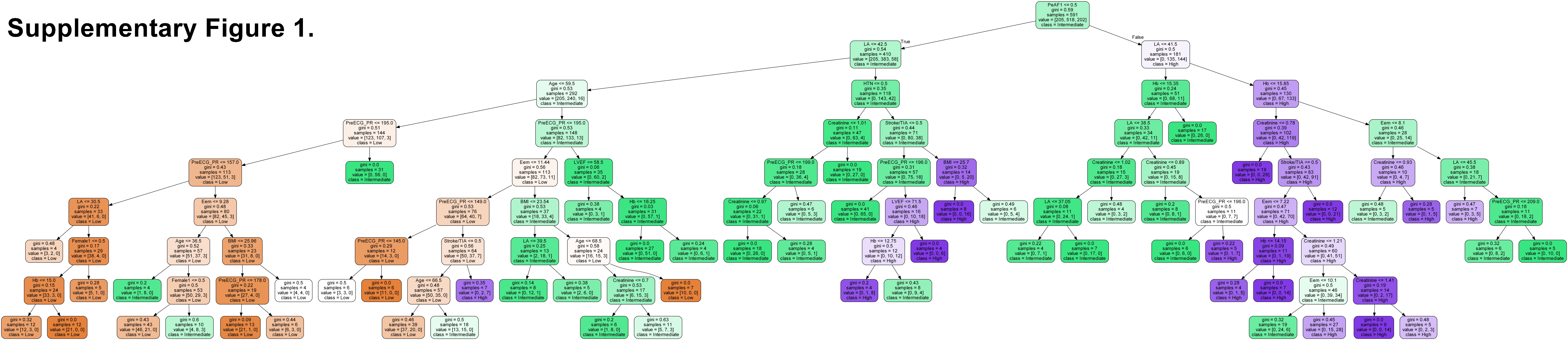

Supplement: Supplementary Figure 1 — Example of a trained first decision tree. [file Image_1.JPEG]

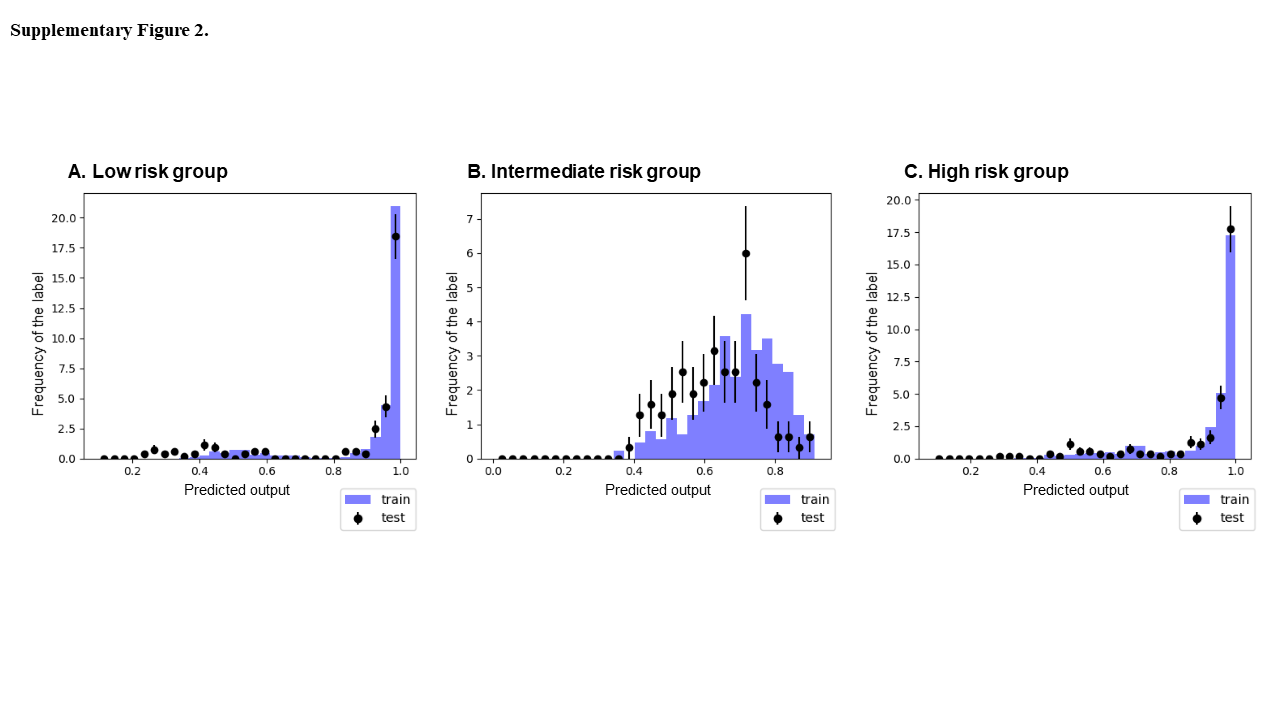

Supplement: Supplementary Figure 2 — Results of overfitting checks for three STAAR groups. (A–C) Each group was divided by a one-vs.-all strategy and identified by the frequency of predicted value in the training and test sets. [file Image_2.tif]

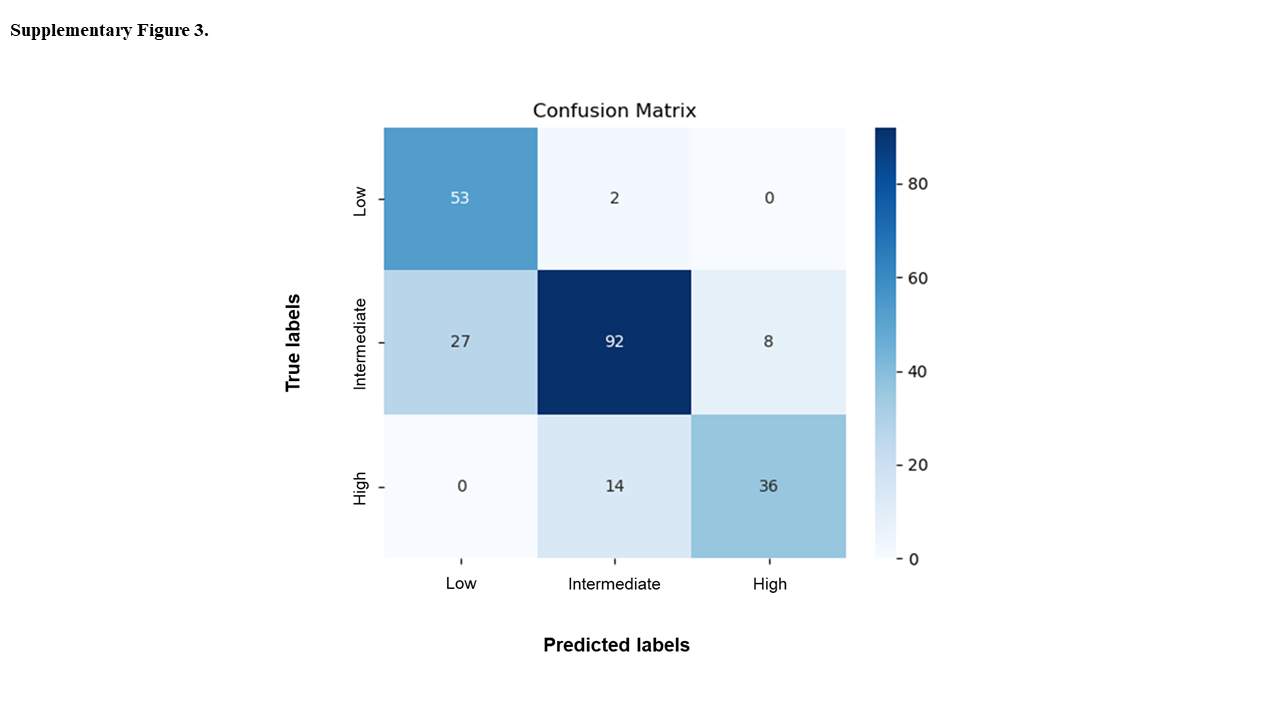

Supplement: Supplementary Figure 3 — Confusion matrix result of artificial intelligence model (Test 4) for three STAAR group. [file Image_3.tif]
