# Supplementary material for: Machine Learning-Predicted Progression to Permanent Atrial Fibrillation After Catheter Ablation
Source: Front Cardiovasc Med. 2022 Feb 16;9:813914. doi: 10.3389/fcvm.2022.813914 (PMC8890475; doi:10.3389/fcvm.2022.813914)
Supplement: Supplementary Table 1 — Cox regression analysis of the pre-procedural variables for the progression to permanent AF. [file Data_Sheet_1.docx]

**Supplementary Table 1. Cox regression analysis of the pre-procedural variables for the progression to permanent AF**

| **Variables** | **Univariate analysis** | | | **Multivariate analysis** | | | |
| --- | --- | --- | --- | --- | --- | --- | --- |
|  | β coefficient | HR (95%CI) | p-value | | β coefficient | HR (95% CI) | p-value |
| Age ≥ 65 years | 0.421 | 1.523 (0.996-2.329) | **0.052** | | 0.145 | 1.156 (0.733-1.822) | 0.533 |
| Female | -0.003 | 0.997 (0.626-1.588) | 0.989 | |  |  |  |
| Persistent AF at the diagnosis | 1.268 | 3.554 (2.335-5.408) | **<0.001** | | 0.509 | 1.663 (1.002-2.760) | **0.049** |
| BMI ≥ 25 kg/m^2^ | 0.558 | 1.748 (1.152-2.652) | **0.009** | | 0.351 | 1.420 (0.904-2.232) | 0.351 |
| CHA_2_DS_2_VASc score | 0.164 | 1.179 (1.041-1.335) | **0.009** | |  |  |  |
| CHF | 0.727 | 2.068 (1.116-3.833) | **0.021** | | 0.216 | 1.241 (0.643-2.394) | 0.519 |
| Hypertension | 0.151 | 1.162 (0.772-1.751) | 0.471 | |  |  |  |
| Diabetes mellitus | 0.226 | 1.253 (0.720-2.180) | 0.425 | |  |  |  |
| Stroke/TIA | 0.804 | 2.235 (1.361-3.670) | **0.001** | | 0.779 | 2.180 (1.305-3.641) | **0.003** |
| Vascular disease | -0.503 | 0.605 (0.292-1.250) | 0.174 | |  |  |  |
| LA dimension ≥ 43 mm | 1.307 | 3.697 (2.334-5.855) | **<0.001** | | 0.714 | 2.042 (1.185-3.520) | **0.010** |
| LVEF, % | -0.014 | 0.986 (0.963-1.010) | 0.254 | |  |  |  |
| EEm | 0.030 | 1.030 (0.988-1.074) | 0.166 | |  |  |  |
| Creatinine, mg/dL | -0.344 | 0.709 (0.323-1.557) | 0.391 | |  |  |  |
| Hemoglobin, g/dL | 0.027 | 1.027 (0.990-1.065) | 0.153 | |  |  |  |
| Pre-ECG PR interval ≥ 196 ms | 1.086 | 2.961 (1.934-4.535) | **<0.001** | | 0.743 | 2.103 (1.346-3.285) | **0.001** |
| Mean LA voltage < 1.109 mV | 1.232 | 3.429 (2.088-5.632) | **<0.001** | | 0.780 | 2.182 (1.274-3.739) | **0.004** |
| Extra-PV LA ablation | 0.125 | 1.133 (0.747-1.719) | 0.555 | |  |  |  |
| BDB of postero-inferior line | -0.057 | 0.944 (0.496-1.796) | 0.861 | |  |  |  |
| CFAE ablation | 0.582 | 1.789 (0.896-3.575) | 0.099 | |  |  |  |

AF=atrial fibrillation, CHF=congestive heart failure, TIA=transient ischemic attack, LA=left atrium, LVEF=left ventricular ejection fraction, Eem = peak transmitral flow velocity (E), and tissue Doppler echocardiography of the peak septal mitral annular velocity (Em), PV=pulmonary vein, BDB=bidirectional block, CFAE=complex fractionated atrial electrogram

**Supplementary Table 2. Prediction model and risk scores for the progression to permanent AF (STAAR score)**

|  | Hazard ratio (95% CI) | β coefficient | P-value | Risk scores |
| --- | --- | --- | --- | --- |
| Previous history of a Stroke or TIA |  |  |  |  |
| No | Reference |  |  |  |
| Yes | 2.180 (1.305-3.641) | 0.779 | 0.003 | 2 |
| Type of AF |  |  |  |  |
| Paroxysmal atrial fibrillation | Reference |  |  |  |
| Persistent atrial fibrillation | 1.663 (1.002-2.760) | 0.509 | 0.049 | 1 |
| Left Atrial dimension |  |  |  |  |
| < 43 mm | Reference |  |  |  |
| ≥ 43 mm | 2.042 (1.185-3.520) | 0.714 | 0.010 | 1 |
| Left Atrial voltage |  |  |  |  |
| ≥ 1.109 mV | Reference |  |  |  |
| < 1.109 mV | 2.182 (1.274-3.739) | 0.780 | 0.004 | 2 |
| PR interval |  |  |  |  |
| < 196 ms | Reference |  |  |  |
| ≥ 196 ms | 2.103 (1.346-3.285) | 0.743 | 0.001 | 1 |

AF=atrial fibrillation, TIA=transient ischemic attack

**Supplementary Table 3.** Optimal hyperparameters selected by grid search.

| **Parameters** | **Selected value** | **Search**  **range** | **Description** |
| --- | --- | --- | --- |
| bootstrap | True | False, True | Whether bootstrap samples are  used when building trees |
| max_depth | 8 | 4 to 10 | The maximum depth of the tree |
| max_features | 5 | 3 to 5 | The number of features to consider when looking for the best split |
| min_samples_leaf | 4 | 3 to 6 | The minimum number of samples  required to be at a leaf node |
| min_samples_split | 10 | 6, 8, 10 | The minimum number of samples  required to split an internal node |
| n_estimators | 100 | 50, 100, 200 | The number of trees in the forest |

**Supplementary Table 4. Linear regression analysis of the pre-procedural variables associated with the STAAR score**

| **Variables** | **Univariate analysis** | |
| --- | --- | --- |
|  | β (95% CI) | P-value |
| Age, years | 0.035 (0.027 - 0.044) | **<0.001** |
| Female | 0.139 (-0.086 - 0.365) | 0.225 |
| Persistent AF at the diagnosis | 2.189 (2.013 - 2.365) | **<0.001** |
| Body mass index, kg/m^2^ | 0.040 (0.009 - 0.071) | **0.012** |
| CHA_2_DS_2_VASc score | 0.378 (0.318 - 0.437) | **<0.001** |
| Congestive heart failure | 0.619 (0.294 - 0.945) | **<0.001** |
| Hypertension | 0.380 (0.182 - 0.577) | **<0.001** |
| Diabetes mellitus | 0.320 (0.038 - 0.602) | **0.026** |
| Stroke/TIA | 2.365 (2.090 - 2.639) | **<0.001** |
| Vascular disease | 0.311 (0.014 - 0.609) | **0.040** |
| LA dimension, mm | 0.143 (0.129 - 0.157) | **<0.001** |
| LVEF, % | -0.021 (-0.033 - 0.010) | **<0.001** |
| EEm | 0.093 (0.070 - 0.116) | **<0.001** |
| Creatinine, mg/dL | 0.310 (0.021 - 0.599) | **0.036** |
| Hemoglobin, g/dL | -0.078 (-0.144 - -0.011) | **0.022** |
| Pre-ECG PR interval, ms | 0.023 (0.020 - 0.026) | **<0.001** |

CI=confidence interval, AF=atrial fibrillation, TIA=transient ischemic attack, LA=left atrium, LVEF=left ventricular ejection fraction, EEm = peak transmitral flow velocity (E), and tissue Doppler echocardiography of the peak septal mitral annular velocity (em), ECG=electrocardiography

**Supplementary Table 5. Baseline characteristics during the *de novo* ablation according STAAR risk groups.**

|  | **Low risk** | **Intermediate risk** | **High risk** | **p-value** |
| --- | --- | --- | --- | --- |
|  | **(n=281)** | **(n=658)** | **(n=275)** |  |
| Age, years | 55.7 ± 11.5 | 58.3 ± 10.8 | 62.6 ± 9.5 | <0.001 |
| Female | 72 (25.6%) | 169 (25.7%) | 81 (29.5%) | 0.457 |
| Persistent AF at diagnosis | 0 (0%) | 175 (26.6%) | 206 (74.9%) | <0.001 |
| Body mass index, kg/m^2^ | 24.4 ± 2.9 | 25.1 ± 3.1 | 25.3 ± 3.6 | 0.002 |
| CHA_2_DS_2_VASc score | 1.1 ± 1.2 | 1.7 ± 1.5 | 2.5 ± 1.8 | <0.001 |
| CHF | 13 (4.6%) | 70 (10.6%) | 43 (15.6%) | <0.001 |
| Hypertension | 102 (36.3%) | 323 (49.1%) | 150 (54.5%) | <0.001 |
| Diabetes mellitus | 26 (9.3%) | 104 (15.8%) | 46 (16.7%) | 0.016 |
| Stroke/TIA | 0 (0%) | 53 (8.1%) | 90 (32.7%) | <0.001 |
| Vascular disease | 21 (7.5%) | 88 (13.4%) | 41 (14.9%) | 0.014 |
| LA dimension, mm | 36.6 ± 3.8 | 41.3 ± 5.8 | 46.1 ± 4.9 | <0.001 |
| LVEF, % | 65.2 ± 6.6 | 63.0 ± 8.6 | 62.0 ± 9.0 | <0.001 |
| EEm (n=1,159) | 8.9 ± 2.9 | 10.2 ± 4.0 | 11.5 ± 5.3 | <0.001 |
| Creatinine, mg/dL | 0.9 ± 0.4 | 0.9 ± 0.3 | 1.0 ± 0.2 | 0.268 |
| Hemoglobin, g/dL | 14.6 ± 1.4 | 14.4 ± 1.5 | 14.3 ± 1.5 | 0.048 |
| Pre-ECG PR interval, ms | 166.5 ± 17.2 | 184.0 ± 28.0 | 202.1 ± 37.2 | <0.001 |

AF=atrial fibrillation, AT=atrial tachyarrhythmia, CHF=congestive heart failure, TIA=transient ischemic attack, LA=left atrium, LVEF=left ventricular ejection fraction, Eem = peak transmitral flow velocity (E), and tissue Doppler echocardiography of the peak septal mitral annular velocity (Em), ECG=electrocardiography

**Supplementary Table 6. Baseline characteristics during the *de novo* ablation procedure in the independent cohort.**

|  | **Overall patients (n=658)** | **Controlled AF/AT** | **Progression to permanent AF** | **p-value** |
| --- | --- | --- | --- | --- |
|  |  | **(n=634)** | **(n=24)** |  |
| Age, years | 56.8 ± 10.7 | 56.7 ± 10.8 | 57.5 ± 9.6 | 0.732 |
| Female | 137 (20.8%) | 134 (21.1%) | 3 (12.5%) | 0.306 |
| Persistent AF at diagnosis | 265 (40.3%) | 243 (38.3%) | 22 (91.7%) | **<0.001** |
| Body mass index, kg/m^2^ | 24.9 ± 3.1 | 24.9 ± 3.1 | 25.6 ± 2.7 | 0.266 |
| CHA_2_DS_2_VASc score | 1.2 ± 1.3 | 1.2 ± 1.3 | 1.0 ± 1.3 | 0.396 |
| CHF | 10 (1.5%) | 10 (1.6%) | 0 (0%) | >0.999 |
| Hypertension | 244 (37.1%) | 235 (37.1%) | 9 (37.5%) | 0.966 |
| Diabetes mellitus | 56 (8.5%) | 53 (8.4%) | 3 (12.5%) | 0.449 |
| Stroke/TIA | 59 (9.0%) | 58 (9.1%) | 1 (4.2%) | 0.714 |
| Vascular disease | 45 (6.8%) | 44 (6.9%) | 1 (4.2%) | >0.999 |
| LA dimension, mm | 41.4 ± 5.8 | 41.2 ± 5.7 | 46.9 ± 7.0 | **<0.001** |
| LVEF, % | 55.2 ± 5.6 | 55.3 ± 5.5 | 51.1 ± 6.9 | **0.008** |
| EEm | 9.0 ± 3.5 | 9.0 ± 3.5 | 8.9 ± 2.7 | 0.868 |
| Creatinine, mg/dL | 1.0 ± 0.6 | 1.0 ± 0.6 | 1.0 ± 0.2 | 0.946 |
| Hemoglobin, g/dL | 15.1 ± 7.6 | 15.1 ± 7.8 | 14.9 ± 1.2 | 0.869 |
| Pre-ECG PR interval, ms | 180.9 ± 39.2 | 179.7 ± 37.8 | 210.5 ± 58.8 | **0.018** |

AF=atrial fibrillation, AT=atrial tachyarrhythmia, CHF=congestive heart failure, TIA=transient ischemic attack, LA=left atrium, LVEF=left ventricular ejection fraction, Eem = peak transmitral flow velocity (E), and tissue Doppler echocardiography of the peak septal mitral annular velocity (Em), ECG=electrocardiography

**Supplementary Table 7. Prediction performance of the ML-prediction model for the three STAAR groups in the development cohort**

|  | AUC | Sens | Spec | PPV | NPV | Gini | Loss | MSE | ACC |
| --- | --- | --- | --- | --- | --- | --- | --- | --- | --- |
| Test 1 |  |  |  |  |  |  |  |  |  |
| LRG | 0.947 | 0.945 | 0.887 | 0.722 | 0.981 | 0.894 | 0.901 | 0.947 | 0.945 |
| IRG | 0.852 | 0.685 | 0.876 | 0.870 | 0.697 | 0.704 | 0.772 | 0.852 | 0.685 |
| HRG | 0.946 | 0.960 | 0.857 | 0.649 | 0.987 | 0.892 | 0.879 | 0.946 | 0.960 |
| Test 2 |  |  |  |  |  |  |  |  |  |
| LRG | 0.896 | 0.927 | 0.842 | 0.646 | 0.974 | 0.791 | 0.862 | 0.896 | 0.927 |
| IRG | 0.786 | 0.661 | 0.800 | 0.800 | 0.661 | 0.572 | 0.724 | 0.786 | 0.661 |
| HRG | 0.946 | 0.920 | 0.857 | 0.639 | 0.975 | 0.892 | 0.871 | 0.946 | 0.920 |
| Test 3 |  |  |  |  |  |  |  |  |  |
| LRG | 0.914 | 0.927 | 0.836 | 0.638 | 0.974 | 0.828 | 0.858 | 0.914 | 0.927 |
| IRG | 0.788 | 0.740 | 0.705 | 0.752 | 0.692 | 0.575 | 0.724 | 0.788 | 0.740 |
| HRG | 0.943 | 0.940 | 0.874 | 0.671 | 0.981 | 0.885 | 0.888 | 0.943 | 0.940 |
| Test 4 |  |  |  |  |  |  |  |  |  |
| LRG | 0.935 | 0.945 | 0.847 | 0.658 | 0.980 | 0.870 | 0.871 | 0.935 | 0.945 |
| IRG | 0.855 | 0.717 | 0.848 | 0.850 | 0.712 | 0.709 | 0.776 | 0.855 | 0.717 |
| HRG | 0.965 | 0.960 | 0.890 | 0.706 | 0.988 | 0.930 | 0.905 | 0.965 | 0.960 |
| Test 5 |  |  |  |  |  |  |  |  |  |
| LRG | 0.923 | 0.927 | 0.836 | 0.638 | 0.974 | 0.846 | 0.858 | 0.923 | 0.927 |
| IRG | 0.828 | 0.724 | 0.800 | 0.814 | 0.706 | 0.656 | 0.759 | 0.828 | 0.724 |
| HRG | 0.950 | 0.900 | 0.846 | 0.616 | 0.969 | 0.901 | 0.858 | 0.950 | 0.900 |

ML=machine learning, LRG=low risk group, IRG=intermediate risk group, HRG=high risk group, AUC=area under the curve, Sens=sensitivity, Spec=specificity, PPV=positive predictive value, NPV=negative predictive value, Gini=gini coefficient, Loss= logit loss, MSE=mean squared error, ACC=accuracy

**Supplementary Table 8. The previous studies and risk model predicting AF recurrence in the patients with repeat ablations for AF**

**(1-9)**

| **Study** | **Risk model** | **Number of predictors for risk model** | **Purpose**  **of study** | **Invasive or non-invasive risk model** | **Number of**  **study population** | **Follow-up**  **duration** | **AUC** |
| --- | --- | --- | --- | --- | --- | --- | --- |
| Wojcik 2013 | ALARMEc | 4 | Development ofrisk model | Non-invasive | n=213 | 24 months | 0.66 |
| Kornej 2017 | APPLE | 5 | Validation of  risk model | Non-invasive | n=379 | 3mo-12 months | 0.62 |
| Jud 2019 | APPLE | 5 | Validation of  risk model | Non-invasive | n=192 | 3mo-24 months  (mean 19 months) | 0.62 |
|  | SUCCESS | 6 | Development of risk model | Invasive | n=192 | 3mo-24months  (mean 19 months) | 0.66 |
| Winkle 2016 | CAAP-AF | 6 | Development of risk model | Non-invasive | n=1125 | Mean 2.5 ± 1.7  years | 0.69 |
|  | CAAP-AF | 6 | Validation of  risk model | Non-invasive | n=937 | Mean 1.8 ± 0.9  years | 0.65 |
| Tang 2012 | HATCH | 5 | Validation of  risk model | Non-invasive | n=488 | Mean 823 ± 532 days | 0.49 |
| Schmidt 2014 | HATCH | 5 | Validation of  risk model | Non-invasive | n=449 | Mean 12.7 ± 7.1 months | Not  available |
| Mujovic 2017 | MB-LATER | 5 | Development of risk model | Invasive | n=133 | Mean 29.1 ± 10.1 months | 0.78 |
|  | MB-LATER | 5 | Validation of  risk model | Invasive | n=39 | Mean 14.6 ± 1.9 months | 0.83 |
|  | APPLE | 5 | Validation of  risk model | Non-invasive | n=133 | Mean 29.1 ± 10.1 months | 0.72 |
|  | ALARMc | 4 | Validation of  risk model | Non-invasive | n=133 | Mean 29.1 ± 10.1 months | 0.67 |
|  | BASE-AF2 | 6 | Validation of  risk model | Invasive | n=133 | Mean 29.1 ± 10.1 months | 0.65 |
|  | HATCH | 5 | Validation of  risk model | Non-invasive | n=133 | Mean 29.1 ± 10.1 months | 0.58 |
| Potpara 2019 | MB-LATER | 5 | Validation of  risk model | Invasive | n=226 | Median 2 years  (IQR 1.1-4.1) | 0.62 |
|  | CAAP-AF | 6 | Validation of  risk model | Non-invasive | n=226 | Median 2 years  (IQR 1.1-4.1) | 0.59 |
| Bavishi 2019 | MB-LATER | 5 | Validation of  risk model | Invasive | n=542 | Median 21.5  months | 0.58 |
|  | BASE-AF2 | 6 | Validation of  risk model | Invasive | n=542 | Median 21.5  months | 0.65 |
|  | APPLE | 5 | Validation of  risk model | Non-invasive | n=542 | Median 21.5  months | 0.54 |
|  | ALARMEc | 4 | Validation of  risk model | Non-invasive | n=542 | Median 21.5  months | 0.49 |
| Current study | STAAR | 5 | Development of risk model | Invasive | n=1214 | Mean 56.2 ± 33.8  Median 51.4  (IQR 26.9, 83.7)  months | 0.80 |

AF=atrial fibrillation, AUC=area under the curve, IQR=interquartile range

**Reference for supplementary Table 8**

1. Wójcik M, Berkowitsch A, Greiss H, Zaltsberg S, Pajitnev D, Deubner N, et al. Repeated catheter ablation of atrial fibrillation: how to predict outcome? Circ J. (2013) 77(9):2271-9. doi:10.1253/circj.cj-13-0308

2. Kornej J, Hindricks G, Arya A, Sommer P, Husser D, Bollmann A. The APPLE Score - A Novel Score for the Prediction of Rhythm Outcomes after Repeat Catheter Ablation of Atrial Fibrillation. PLoS One. (2017) 12(1):e0169933. doi:10.1371/journal.pone.0169933

3. Jud FN, Obeid S, Duru F, Haegeli LM. A novel score in the prediction of rhythm outcome after ablation of atrial fibrillation: The SUCCESS score. Anatol J Cardiol. (2019) 21(3):142-9. doi:10.14744/AnatolJCardiol.2018.76570

4. Winkle RA, Jarman JW, Mead RH, Engel G, Kong MH, Fleming W, et al. Predicting atrial fibrillation ablation outcome: The CAAP-AF score. Heart Rhythm. (2016) 13(11):2119-25. doi:10.1016/j.hrthm.2016.07.018

5. Tang RB, Dong JZ, Long DY, Yu RH, Ning M, Jiang CX, et al. Efficacy of catheter ablation of atrial fibrillation beyond HATCH score. Chin Med J (Engl). (2012) 125(19):3425-9.

6. Schmidt EU, Schneider R, Lauschke J, Wendig I, Bänsch D. The HATCH and CHA2DS 2-VASc scores. Prognostic value in pulmonary vein isolation. Herz. (2014) 39(3):343-8. doi:10.1007/s00059-013-3835-x

7. Mujović N, Marinković M, Marković N, Shantsila A, Lip GY, Potpara TS. Prediction of very late arrhythmia recurrence after radiofrequency catheter ablation of atrial fibrillation: The MB-LATER clinical score. Sci Rep. (2017) 7:40828. doi:10.1038/srep40828

8. Potpara TS, Mujovic N, Sivasambu B, Shantsila A, Marinkovic M, Calkins H, et al. Validation of the MB-LATER score for prediction of late recurrence after catheter-ablation of atrial fibrillation. Int J Cardiol. (2019) 276:130-5. doi:10.1016/j.ijcard.2018.08.018

9. Bavishi AA, Kaplan RM, Peigh G, Diaz CL, Baman JR, Trivedi A, et al. Patient characteristics as predictors of recurrence of atrial fibrillation following cryoballoon ablation. Pacing Clin Electrophysiol. (2019) 42(6):694-704. doi:10.1111/pace.13669
